# Protective effect of resveratrol against pressure overload-induced heart failure

**DOI:** 10.1002/fsn3.92

**Published:** 2014-03-05

**Authors:** Prakash K Gupta, Donald J DiPette, Scott C Supowit

**Affiliations:** 1 Department of Cell Biology & Anatomy, University of South Carolina School of MedicineColumbia, South Carolina; 2 Department of Medicine, University of South Carolina School of MedicineColumbia, South Carolina

**Keywords:** Cardiac remodeling, heart failure, oxidative stress, pressure overload, resveratrol

## Abstract

Transverse aortic constriction (TAC)-induced pressure overload (PO) causes adverse cardiac remodeling and dysfunction that progresses to heart failure (HF). The purpose of this study was to determine whether the potent antioxidant, resveratrol, significantly attenuates PO-induced HF in wild-type mice. Male C57BL6 mice were subjected to either sham or TAC surgery. One group of TAC mice was given daily resveratrol treatment. Echocardiographic, biometric, and immunohistological analyses were performed on the three groups of mice. All echocardiographic parameters demonstrated significantly greater adverse cardiac remodeling and dysfunction in the TAC compared to the sham mice. Increases in the ratios of heart weight (HW)/body weight (BW) and lung weight (LW)/BW and a sharp decline in the percentage of ejection fraction and fractional shortening were found in TAC relative to sham mice. Likewise, the TAC protocol increased markers of oxidative stress, cardiac hypertrophy, inflammation, fibrosis, hypoxia, and apoptosis. These pathological changes were significantly attenuated by resveratrol treatment. Resveratrol treatment significantly attenuates the adverse cardiac remodeling and dysfunction produced by the TAC protocol in C57/BL6 mice and this activity is mediated, at least in part, by the inhibition of oxidative stress and inflammation indicating a therapeutic potential of resveratrol in HF.

## Introduction

Heart failure (HF) is a primary cause of morbidity and mortality in many parts of the world. (Wu et al. 2002; Liew and Dzau 2004). HF is a multicausal chronic syndrome in which the heart fails to work efficiently and is unable to meet the metabolic demands of tissues (Juric et al. 2007). Diseases such as hypertension, valvular heart disease, ischemic heart disease, or cardiomyopathy subsequently lead to HF (Gomez-Arroyo et al. 2013). HF is preceded by left ventricular hypertrophy in response to pressure overload (PO). Left ventricular hypertrophy compensates for the PO, however, adverse remodeling impairs left ventricular function. Although cardiac hypertrophy is an adaptation that is beneficial to the stressed heart in the initial stages wherein cardiomyocytes enlarge in size to achieve adequate function in the presence of chronic pathological stress (Frey and Olson 2003) this compensatory phase is temporary because in the face of continued stress the heart eventually enters into a decompensatory stage (Adler et al. 2011). This transition from compensatory to decompensatory stage is characterized by marked increases in cardiac fibrosis, hypoxia, and apoptosis that lead to irreversible functional changes and HF (Juric et al. 2007; Wojciechowski et al. 2010; Yu and Li 2010). Although current pharmacological therapies have proven reasonably effective for patients with cardiac hypertrophy and early stages of HF, in many people these agents do not prevent the progression to moderate to severe HF with the ensuing increase in morbidity and mortality (Tunuguntla 2007; De Luca et al. 2008; Tavares et al. 2008). Therefore, it is imperative to explore alternate strategies to provide safe and effective treatment for HF.

A number of in vivo and in vitro studies have yielded substantial evidence indicating that oxidative stress and inflammation play a central role in the pathogenesis of a number of cardiovascular diseases including HF (Giordano 2005; Murdoch et al. 2006; Takimoto and Kass 2007; Valko et al. 2007). Endogenous antioxidant defense systems regulate the levels of reactive oxygen species to maintain normal physiological homeostasis. However, in a significant number of pathological conditions, including various cardiovascular disease states, reactive oxygen species production prevails over the antioxidant defense mechanisms leading to chronic oxidative stress thus causing injury to cellular components. (Movahed et al. 2012).

Several plant and fruit extracts containing phytochemicals have been screened for treating a variety of pathophysiological conditions, including cardiovascular diseases (Ardiansyah et al. 2008; Seymour et al. 2008). Resveratrol (*trans*-3′,4′,5-trihydroxystilbene), a polyphenol predominantly found in grapes and berries, or its analogs have been studied in different experimental settings of hypertension, myocardial infarction, and HF (Thandapilly et al. 2010; Wojciechowski et al. 2010). Resveratrol has been shown to have potent antioxidative, antiapoptotic, and anti-inflammatory properties (Kalra 2003; De Caterina et al. 2006; Lagouge et al. 2006; Ramaa et al. 2006; Pfluger et al. 2008; Thandapilly et al. 2010; Wojciechowski et al. 2010; Rimbaud et al. 2011; Movahed et al. 2012). However, no study has examined the effects of resveratrol in preventing chronic pathological changes in cardiac structure and function in PO-induced HF in C57/BL6 mice, the genetic background for most genetically modified mice which should provide a useful tool to elucidate the mechanisms underlying the cardioprotective activities of this phytochemical.

## Materials and Methods

### Ethics statements

All experiments were performed in conformity with the National Institutes of Health *Guidelines for the Care and Use of Laboratory Animals* guiding principles in the care and use of animals. Authorizations to conduct animal experiments were approved by the University Animal Care and Use Committee of the University of South Carolina.

### Pressure overload model

Eight to ten-week-old (26–28 g) C57/BL6 male mice were used in this study (Harlan Sprague, Dawley). Surgical details of the transverse aortic constriction (TAC) procedure are described elsewhere (Wojciechowski et al. 2010). Three groups (*n* = 6/group) of mice were used for this study. Samples from every mouse used in this study were included for each assay described in this section. Briefly, mice were anesthetized with 5% isoflurane and maintained with 2% isoflurane. A midline thoracotomy at the level of the suprasternal notch was performed allowing direct visualization of the transverse aorta. Using a 27-gauge needle and 7-0 silk suture, transverse aorta was banded yielding 70–80% constriction. Sham operations on age-matched mice only omitted the actual aortic banding. Twenty-four hours post surgery, one group of aortic-banded mice were administered resveratrol (10 mg·kg body weight^−1^·day^−1^) by oral gavage for the duration of the protocol (28 days). An equivalent volume of water as a placebo was given to a second group of TAC mice and the sham-operated mice. Systemic blood pressures were not determined since the TAC procedure does not increase the blood pressure distal to the constriction site (Wu et al. 2002). A feasibility study was performed using two animals per for the three groups described above (sham operated, WT TAC, and WT TAC with resveratrol treatment). A fourth group was sham operated with resveratrol treatment. Echocardiographic parameters were determined as described below. The results were consistent with those obtained from the complete study (*n* = 6/group). Moreover, the sham-operated + resveratrol data were not different from the sham operated alone so the former group was not included in the full study.

### Assessment of cardiac structure and function

Two-dimensional-guided M-mode echocardiography of the mice was performed on days 0, 7, 14, 21 and 28, using a Vevo 770 High-Resolution Imaging System with a 37.5-MHz high-frequency linear transducer (VisualSonics Inc., Toronto, ON, Canada) as described previously (Wojciechowski et al. 2010). Briefly, mice were anesthetized with 3% isoflurane and maintained with 1.5% isoflurane supplemented with 100% O2. A guided M-mode echocardiogram was recorded through the anterior and posterior LV walls at 21 frames/s. Images were obtained at the level of the papillary muscle tips, and measurements were then performed to obtain the LV internal dimension (LVID; in mm), LV posterior wall thickness (LVPW; in mm). LV percent fractional shortening (FS %) and ejection fraction (EF %) were calculated via VisualSonics Measurement Software.

### Isolation of mouse hearts

After the 28 day protocol, the mice were weighed and anesthetized by using isoflurane before being euthanized. Hearts and lungs were removed and washed in ice-cold saline and the wet weight of the heart and wet weight of the lung were measured. Tissue was separated, flash-frozen in liquid nitrogen, and subsequently stored at −85°C until further experimentation.

### Preparation of the LV homogenates

LV tissue was pulverized and homogenized in a buffer containing 10 mmol/L NaHCO_3_, 5 mmol/L NaN_3_, and 15 mmol/L Tris·HCl at pH 6.8 (10 mL/g tissue). This was aliquoted and frozen in liquid nitrogen before storage at −85°C. The buffer used for LV tissue homogenization also contained a cocktail of protease inhibitors consisting of (in *μ*mol/L) 1 leupeptin, 1 pepstatin, and 100 phenylmethylsulfonyl fluoride.

### Histological and immunochemical analyses

Hearts were excised and washed with 0.9% saline, fixed in 10% formalin, and embedded in paraffin. Sections were cut (5 *μ*m, Leica RM2030, rotary microtome, Wetzlar, Germany) and stored at room temperature until staining. For left ventricular cardiomyocyte cross-sectional area, sections were deparaffinized and stained for membranes with Texas Red-X conjugated wheat germ agglutinin (WGA) (Invitrogen Corp., Carlsbad, CA) and observed under the fluorescence microscope (Nikon Eclipse E600; Nickon Inc., Melville, NY) at 400× magnification. Twenty fields of each section were randomly photographed using Qcapture software (MAG Corp., Pleasanton, CA) and cardiomyocyte area was measured using Image-Pro Plus software (Media Cybernetics, Inc., Bethesda, MD). For myocardial fibrosis, sections were stained for collagen with a Masson's Trichrome Kit (Poly Scientific, Bay Shore, NY) according to the protocol provided by the manufacturer. Twenty fields of each section were randomly photographed and observed under light microscope (Nikon Optiphot-2; Nikon Inc.) at 200× magnification. The relative fibrotic area (% of total area) was averaged from 20 sections (Nikon Optiphot-2; Nikon Inc.). Sections were stained with Mac-2 primary antibody (cedarlane, NC) staining kit (Immunocruz ABC staining system; Santa Cruz Biotechnology, Inc., Santa Cruz, CA) for macrophage and Toluidine blue O (Sigma Aldrich, St. Louis, MO) was used for mast cell staining according to the protocol provided by the manufacturer. Sections were observed under light microscope (Nikon Optiphot-2; Nikon Inc.) at 200× magnification. Twenty fields of each section were randomly photographed using Axio Vision 3.1 software (Carl Zeiss Inc., Maple Grove, MN). The number of (the blue stained cells) was counted by Image-Pro Plus software (Media Cybernetics Inc., Rockville, MD). Apoptosis was measured by TUNEL assays on tissue sections using In Situ Cell Death Detection Kit, TMR red (Roche Applied Science, Indianapolis, IN) as described previously (Xing et al. 2012). The apoptotic nuclei were labeled with TUNEL (red) all nuclei were counterstained with DAPI (blue), and cardiomyocyte was stained with antitropomyocin I (green). Images were acquired by (Nikon Eclipse E600; Nickon Inc.) at 400× magnification. Staining of 4-hydroxy-2-nonenal (4-HNE), a marker of lipid peroxidation, and 8-hydroxydeoxyguanosine (8-OHdG), a marker of DNA oxidization, were performed with a mouse anti 4-HNE antibody (ab48506; Abcam Inc., Cambridge, MA) and mouse anti 8-OHdG antibody (sc-660369; Santa Cruz Biotechnology, Inc.), respectively. Cardiomyocytes were stained with a rabbit anti-Tropomyosin I (ab55915; Abcam Inc.). Immunofluorescent staining was performed according to a standard protocol provided by Santa Cruz Biotechnology, Inc. Images were acquired using Qcapture software (MAG Corp., and Nikon Eclipse E600; Nickon Inc.) at 400× magnification and measured using Image-Pro Plus software (Media Cybernetics Inc.)

### Western blotting and protein activity

The enzymatic activity of superoxide dismutase (SOD) was measured by a SOD assay kit (Sigma, Switzerland) and glutathione content was measured by GSH-Glo Glutathione assay kit (Promega, Madison, WI) according to protocols provided by the companies. A 20/20 luminometer (Turner BioSystems, Sunnyvale, CA) was used to detect total glutathione levels while a Spectra Max Plus (Sunnyvale, CA) was used to detect the total SOD activity. Myocardial protein content of cleaved caspase-3 (Santa Cruz Biotechnology), hypoxia-inducible factor 1alpha (Hif-1*α*) (Novus Biologicals, Littleton, CO) from LV homogenates were determined by Western blot analysis as described previously (Zhang et al. 2007). Briefly, total protein from LV tissue was extracted by T-PER tissue protein extraction reagent (Thermo Scientific, Rockford, IL). Protein samples (25–30 *μ*g) were fractionated by sodium dodecyl sulfate-polyacrylamide gel electrophoresis (SDS-PAGE), and then transferred to nitrocellulose membranes. The membranes were probed with corresponding primary antibodies. Appropriate HRP-conjugated secondary antibodies were used and the antibody-antigen complexes in all membranes were detected by the ECL PLUS Detection Kit (Thermo Scientific). The expression of these proteins was quantified with Scion Image (NIH, Bethesda, MD) and adjusted to *β*-actin.

### Statistical analysis

Data were expressed as mean ± SD. Differences among groups were tested by one-way analysis of variance followed by Bonferroni's multiple comparison post hoc test. *P* < 0.05 was considered significantly different.

## Results

### Assessment of body, heart, and lung weights

**Figure 1:**
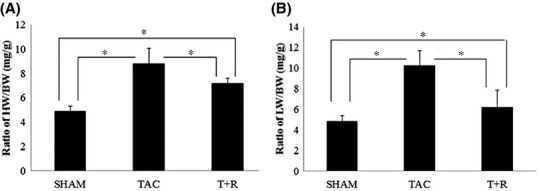
Resveratrol treatment attenuates TAC-induced cardiac hypertrophy and lung edema. Mice were subjected to sham surgery, TAC, or TAC + resveratrol treatment. (A) Ratio of heart weight to body weight: (HW/BW). (B) Ratio of lung weight to body weight (LW/BW). Values are expressed as the mean ± SD. *P < 0.05 was considered statistically significant.

There were no operative deaths within 24 h after the TAC or sham surgeries and there was no significant increase in mortality in any of the three groups. Analysis of mice after 4 weeks of PO showed that heart weight (HW) to body weight (BW) ratios were significantly greater in TAC mice compared to sham-operated mice (Fig. 1A). Similarly, the ratio of LW to BW was also significantly higher in TAC mice compared to sham mice (Fig. 1B) The increases in HW/BW and LW/BW ratios after TAC were significantly attenuated by resveratrol treatment (Fig. 1A and B).

### Resveratrol treatment improves cardiac remodeling and function

**Figure 2:**
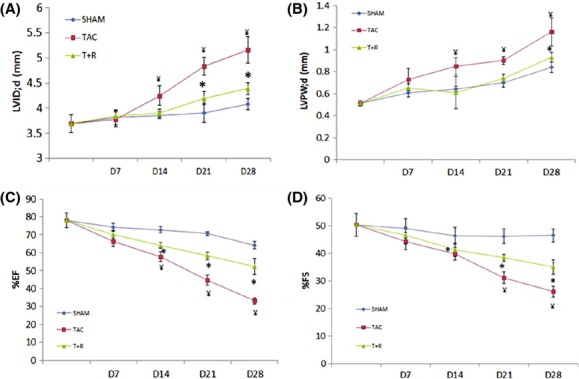
Resveratrol treatment attenuates the adverse cardiac remodeling and contractile dysfunction in TAC-induced heart failure. Echocardiography was performed at the indicated time points in the sham, TAC, and TAC + resveratrol groups of mice. (A) LVID;d. (B) LVPW;d. (C) EF. (D) FS. Values are expressed as the mean ± SD. *P < 0.05 was considered statistically significant.

After 4 weeks of TAC, LVID;d was significantly increased in TAC mice compared to sham operated. Resveratrol treatment was able to significantly attenuate this increase (Fig. 2A). Similarly, LVPW;d was significantly increased in TAC mice compared to sham-operated mice (Fig. 2B) and significantly attenuated by the resveratrol treatment. As shown in Figure 2C and B. LV systolic function, as assessed by EF (Fig. 2C) and FS (Fig. 2D) was significantly decreased in the TAC mice compared to sham-operated mice. Resveratrol treatment significantly attenuated the sharp decline of both parameters.

### Effect of resveratrol on cardiac hypertrophy and fibrosis after TAC

**Figure 3:**
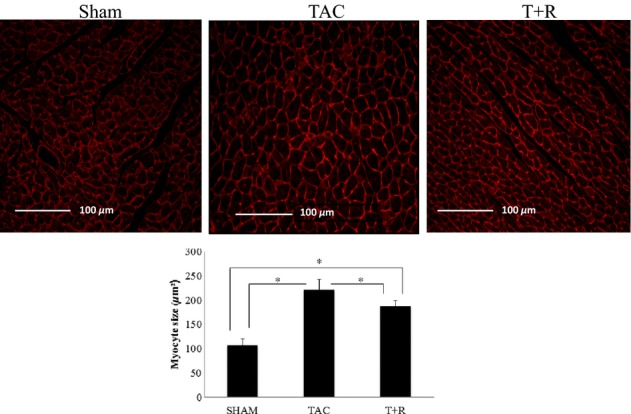
Resveratrol attenuates TAC-induced cardiomyocyte hypertrophy. Tissue slices (LV, day 28) from sham surgery, TAC, and TAC + resveratrol-treated mice were stained with WGA (top panels) and quantified (bottom panel) as described in the Materials and Methods section. Values are expressed as the mean ± SD. *P < 0.05 was considered statistically significant.

**Figure 4:**
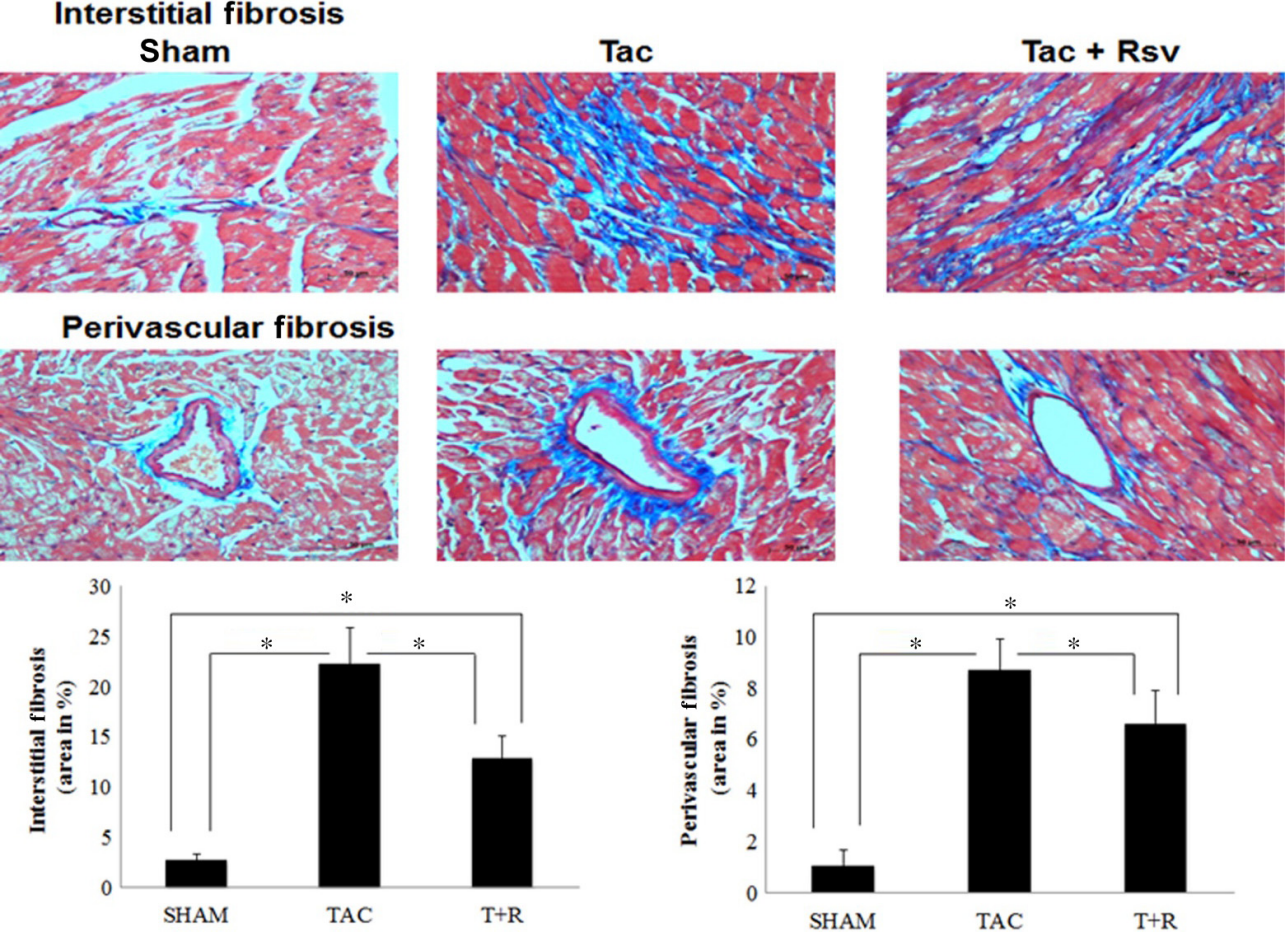
Resveratrol treatment attenuates TAC-induced cardiac fibrosis. The top (interstitial fibrosis) and middle panels (perivascular fibrosis) show representative tissue sections (LV, day 28) from sham, TAC, and TAC + resveratrol mice that were stained for collagen and quantified (bottom panels) as described in the Materials and Methods section. Values are expressed as the mean ± SD. *P < 0.05 was considered statistically significant.

LV hypertrophy assessed by WGA staining was significantly greater in TAC mice compared to sham mice and was significantly attenuated by resveratrol treatment (Fig. 3). Similarly, as shown in Figure 4, LV interstitial and perivascular fibrosis as determined by Masson's trichrome staining was found to be significantly higher in TAC mice compared to sham mice. However, this marked increase in fibrosis was significantly attenuated in the resveratrol-treated group.

### Anti-inflammatory effects of resveratrol

**Figure 5:**
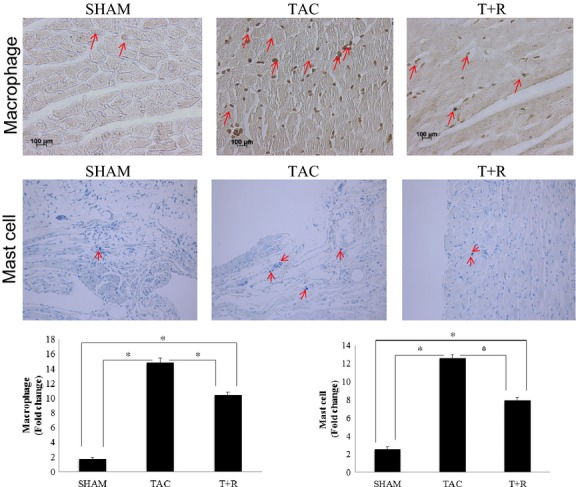
Resveratrol attenuates TAC-induced macrophage and mast cell infiltration. Representative image showing immunohistochemical staining of macrophages (brown color, top panels) and mast cells (blue color, middle panels) from LV sections (28 day) of sham, TAC, and TAC + resveratrol-treated mice. Quantification of the data, as described in the Materials and Methods section, is shown in the bottom panels. Values are expressed as the mean ± SD. *P < 0.05 was considered statistically significant.

As shown in Figure 5 (top and bottom panels) LV macrophage infiltration was significantly increased after 4 weeks of TAC compared to sham-operated mice. Treatment with resveratrol significantly attenuated this inflammatory response when compared to TAC alone. Similarly, Figure 5 (middle and bottom panels) demonstrates that PO significantly increased the number of LV mast cells compared to sham-operated mice. However, treatment with resveratrol significantly attenuated mast cell infiltration compared to TAC alone.

### Resveratrol suppresses PO-induced cardiac oxidative stress

**Figure 6:**
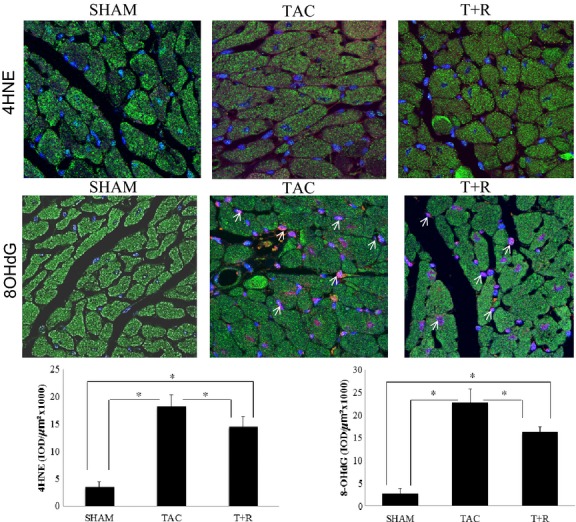
Resveratrol attenuates TAC-induced oxidative stress. Representative images of 4-HNE (top panels) and 8OHdG (middle panels) staining of LV sections at day 28 from sham surgery, TAC, and TAC + resveratrol-treated mice. Areas shown in red are positive for 4-HNE and 8OHdG. Cardiomyocytes were stained green using antitropomyosin I and nuclei in blue were labeled with DAPI. Quantification of the data as described in the Materials and Methods section is shown in the bottom panels. Values are expressed as the mean ± SD. *P < 0.05 was considered statistically significant.

The antioxidative properties of resveratrol were assessed by measuring LV expression of 4-hydroxynonenal (4-HNE, Fig. 6 top and bottom panels), a marker of lipid peroxidation, and 8-OHdG (Fig. 6 middle and bottom panels), a marker of DNA damage induced by oxidative stress. The biomarkers 4-HNE was significantly increased in the mice that underwent TAC procedure relative to the sham surgery mice. Resveratrol treatment significantly attenuated the upregulation of 4-HNE in the resveratrol-treated group. Similarly, 8-OHdG levels were markedly upregulated in the TAC mice compared to their sham counterparts. Again, treatment with resveratrol was able to significantly attenuate this pathological marker of oxidative DNA damage.

### Resveratrol inhibits cardiomyocyte apoptosis

**Figure 7:**
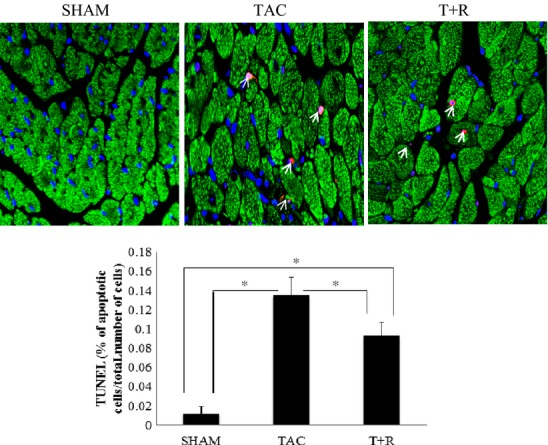
Resveratrol attenuates TAC-induced apoptosis. Representative images of TUNEL staining of LV sections (28 days) from sham surgery, TAC, and TAC + resveratrol treatment are shown in upper panel. Apoptotic nuclei are stained red, cardiomyocytes green (antitropomyosin I) and nuclei blue (DAPI). Values are expressed as the mean ± SD. *P < 0.05 was considered statistically significant.

**Figure 8:**
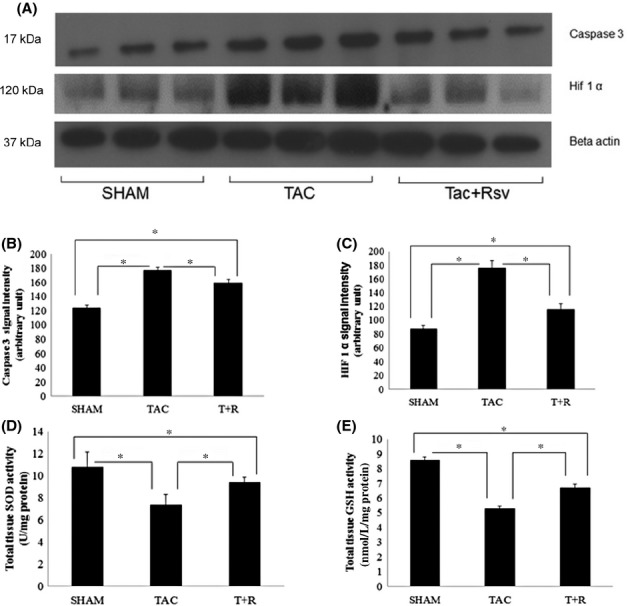
Resveratrol treatment alters pathways that regulate apoptosis, hypoxia, and oxidative stress. (A) Representative composite Western blots of cleaved caspase 3, and HIF1*α* from LV protein extracts of sham surgery, TAC, and TAC + resveratrol treatment (28 day). (B) Quantification of cleaved caspase 3. (C) HIF1*α* protein levels. (D) SOD activity. (E) Glutathione content. All values were normalized to *β*-actin levels. Values are represented as mean ± SD. *P < 0.05 was considered statistically significant.

Terminal deoxynucleotide transferase dUTP nick end-labeling (TUNEL) immunohistochemical staining along with the Western blot for cleaved caspase-3 was performed to assess LV apoptotic death of cardiomyocytes. As shown in Figure 7, the percentage of apoptotic nuclei was significantly greater in the TAC mice compared to sham-operated mice. In line with the TUNEL assay, LV levels of cleaved caspase-3 were significantly upregulated in the TAC mice compared to the sham group (Fig. 8A). As expected, the number of apoptotic nuclei and the production of cleaved caspase-3 were significantly inhibited in the resveratrol-treated mice.

### Resveratrol protects against PO-induced hypoxia

In addition, we analyzed LV expression of the hypoxic factor HIF-1*α*. In the TAC WT hearts there was a significant increase in HIF-1*α* levels as compared to sham-operated mice (Fig. 8A and C). Treatment with resveratrol significantly inhibited HIF-1*α* levels.

### Resveratrol increases antioxidative stress mechanisms

The activity of SOD and levels of glutathione were determined in LV homogenates. SOD activity (Fig. 8D) and the levels of the reactive oxygen species scavenger glutathione (Fig. 8E) were markedly downregulated in TAC mice compared to the sham group. Resveratrol significantly inhibited this decline in SOD activity and glutathione content.

## Discussion

In the last decade, there has been a dramatic increase in the research regarding the protective and therapeutic effects of resveratrol in cardiovascular diseases (Juric et al. 2007; Movahed et al. 2012). Prevention and regression of cardiac structural and functional alterations including pathological hypertrophy by resveratrol treatment in experimental models of PO-induced HF in the rat have been reported (Li et al. 2005; Juric et al. 2007). Therefore, the purpose of this study was to assess the cardioprotective effects of resveratrol in the C57/BL6 mouse PO model to facilitate future studies using genetically modified mice in order to elucidate the mechanisms that mediate the therapeutic activities of this drug. The significant findings of this study were that resveratrol treatment resulted in (1) significant attenuation of adverse cardiac remodeling and dysfunction; (2) significant attenuation of cardiac oxidative stress, inflammation, fibrosis, and apoptosis.

Following initiation of the 28 day TAC protocol the mice developed compensatory cardiac hypertrophy followed by HF as evidenced by LV dilation, reduced FS and EF, and increased LW to BW ratios. Daily resveratrol treatment throughout the course of the protocol significantly improved, but did not prevent, all the pathophysiological structural, functional, and cellular/biochemical parameters examined. To the best of our knowledge, this is the first reported demonstration of the beneficial effects of resveratrol in PO-induced HF in mice. These results are consistent with the study by Juric et al. (2007), which reported that resveratrol had potent cardioprotective effects in aortic-banded rats, as well as a second study also performed in aortic-banded rats that used isorhapontigenin, an analog of resveratrol (Li et al. 2005). Our results are also in agreement with a study demonstrating that resveratrol treatment prevented the cardiac fibrosis and decreased cardiovascular function in deoxycorticosterone-salt (DOC-salt) hypertensive rats. Similarly, resveratrol improved the survival, and cardiac hemodynamics and energetics in the Dahl salt-sensitive model of hypertension (Rimbaud et al. 2011).

Consistent with the studies in the various rat models of HF, resveratrol was very effective in attenuating the PO-induced cardiac hypertrophy and cardiac contractile function in the mouse. Many of the beneficial effects of resveratrol in HF are mediated through the attenuation of oxidative stress which in turn triggers a robust and chronic inflammatory response (Ruetten et al. 2006; DeMarco et al. 2010; Li et al. 2012). In this study, resveratrol treatment significantly attenuated TAC-induced macrophage and mast cell infiltration. Resveratrol not only modulates biochemical responses of polymorphonuclear leukocytes by interfering with the release of inflammatory mediators but also suppresses the activity of macrophages as well as inhibit their migration into the cardiac tissue (Sharma et al. 2007). This response has been shown to be mediated by multiple pathways (Leiro et al. 2005; Kang et al. 2009; Karlsen et al. 2010; Li et al. 2012). Similarly, mast cells have been implicated in the pathogenesis of HF and play a critical role in the fibrotic response to infectious and inflammatory stimuli (Matsumori et al. 1994). Although found mainly in the skin, gastrointestinal tract, and airways, they are known to reside in cardiac tissue and have been shown to play a key role in the pathogenesis of HF (Mina et al. 2013).

Consistent with the enhanced inflammatory response in the TAC mice compared to their WT counterparts, we also found a striking increase in both interstitial and perivascular fibrosis in the PO hearts. This is most likely the primary cause of the significant deterioration of cardiac function. These data clearly indicates that the PO-induced fibrosis seen in the TAC mice is markedly increased compared to sham surgery and is markedly attenuated by resveratrol treatment. There is a significant increase in LV apoptosis in the TAC mice compared to the sham mice which again is significantly reduced by resveratrol. While myocyte apopotosis is well documented in HF and can reduce the force-generating capacity of the myocardium (Juric et al. 2007), apoptotic cells are scattered across the wall of the chamber and are usually found as single cell losses. Although we did not directly examine necrotic cell death in this study, it should be noted that replacement fibrosis in HF occurs in areas of multiple large and diffuse foci that contain a much larger number of dead myocytes as opposed to that seen in apoptosis. While necrosis is not as well studied as apoptosis, it is now clear that it is a tightly regulated process. In HF, it appears that necrotic cell death is triggered at the level of the mitochondria by multiple factors including sympathetic and Ca^2+^ overload, oxidative and metabolic stress, and hypoxia (Schmidt et al. 2002). Several lines of evidence indicate that resveratrol has significant antiapoptotic activity, both in vivo and in vitro, that is mediated by the inhibition of NF-*κ*B, p53 and PGC-1*α* via activation of SIRT1 pathways which stimulate survival pathways (Vaziri et al. 2001; Yeung et al. 2004; Rodgers et al. 2005).

In C57/BL6 mice PO-induced cardiac hypertrophy leads to a mismatch between number of capillaries and the size of cardiomyocytes which results in cardiac hypoxia. We have clearly demonstrated this mismatch in a previous study (Li et al. 2013). As a compensatory response, HIF1*α* and vascular endothelial growth factor have shown to be upregulated during PO-induced hypertrophy in WT hearts (Wang and Si 2013). Indeed, our results with HIF1*α* are consistent with this report. The decrease in cardiac HIF1*α* content in the resveratrol-treated mice indicates that the resveratrol-mediated decrease in cardiac hypertrophy decreases the need for increased vascularization. Other investigators have demonstrated that in addition to inhibiting cardiac hypertrophy, resveratrol supported cardiac microvessel development in cardiac hypertrophic growth, thereby protecting cardiomyocytes against hypertrophic hypoxia-induced injury and death (Li et al. 2013; Wang and Si 2013).

As described previously, perhaps the key cardioprotective activity of resveratrol in the context of HF is through inhibition of oxidative stress. Although resveratrol has direct antioxidant activity, these effects are weak. It is likely that the protective activity of this drug against oxidative stress is mediated through the upregulation of endogenous antioxidant systems (Hung et al. 2002; Cao and Li 2004; Guha et al. 2009). As described previously, we examined SOD activity and GSH levels which act to significantly inhibit oxidative stress (Forstermann 2010). Both SOD activity and GSH levels were markedly reduced in the heart of the TAC mice compared to the controls. Resveratrol treatment attenuated the decrease is both of these antioxidant systems but did not normalize them to levels observed in the sham hearts suggesting that other endogenous antioxidant systems may be involved. Although the exact mechanism of action of resveratrol in mediating these antioxidants remains largely unknown, recent studies have shown that overexpression of SIRT1 leads to upregulation of SOD (Ungvari et al. 2009). Moreover, during oxidative stress, the LKB1/AMPK pathway has been shown to be highly regulated and lipid peroxidation products such as 4-HNE are also elevated. (Dolinsky et al. 2009). AMPK (and its upstream kinase LKB1) not only antagonizes the hypertrophic response but also delays the transition from hypertrophy to HF. AMPK inhibits cardiac remodeling by preventing angiotensin II-induced myocardial fibrosis (Dolinsky et al. 2009). 4-HNE forms covalent adducts with LKB1 leading to LKB1/AMPK signaling inhibition and induction of Mtor/p70s6 kinase-mediated protein synthesis and cardiac myocyte growth. Resveratrol treatment prevents left ventricular hypertrophy by preventing 4-HNE modification of LKB1/AMPK signaling which blunts the prohypertrophic p70S6 kinase response (Ungvari et al. 2009). Consistent with the abovementioned study, our results show a marked induction of 4-HNE level in TAC mice compared to sham operated. However, this upregulation of 4-HNE level was blunted significantly with resveratrol treatment.

In the failing heart, decompensation and cardiomyocyte deficiency is the predominant problem. Under such conditions, an active DNA repair process instead of inhibited protein synthesis is potentiated by resveratrol, which preserves the genomic stability of cardiomyocytes. Oxidative DNA damage in cardiomyocytes was assessed to determine extent of oxidative stress in PO hearts. Consistent with other findings (Sin et al. 2013), the elevation of 8-OHdG in TAC hearts indicated the presence of significant oxidative DNA damage compared to controls. Resveratrol was able to markedly blunt this increase in oxidative stress-induced DNA damage. This finding is consistent with the report of Juric et al. (2007) that demonstrated that the base excision repair pathway, apurinic/apyrimidinic endonuclease redox factor 1 (1) was significantly decreased in TAC rats. However, resveratrol treatment restored the activity of this DNA repair pathway.

In summary, this study demonstrates that resveratrol has potent cardioprotective activities against TAC-induced HF in the C57/BL6 mouse that is mediated through multiple mechanisms and supports further that resveratrol may be a new therapeutic agent in preventing the progression of HF. In addition, the use of genetically modified mice should prove to be a useful tool in elucidating the mechanisms that mediate this effect.

## Clinical Perspective

Although there are no clinical trial studies that directly address the use of resveratrol in HF and there are some questions regarding bioavailability differences of this drug between humans and rodents (Kroon et al. 2010), this study demonstrates the therapeutic potential of resveratrol in the setting of PO-induced HF by maintaining cardiac function through the attenuation of cardiac hypertrophy, fibrosis, inflammation, and subsequent apoptosis induced by oxidative stress hence preventing overt HF. Therefore, these data indicate that resveratrol (or resveratrol analogs) may be a novel therapeutic agent for the treatment of HF.
